# Incidence of prostate, breast, lung and colorectal cancer following new consultation for musculoskeletal pain: A cohort study among UK primary care patients

**DOI:** 10.1002/ijc.28055

**Published:** 2013-01-25

**Authors:** Kelvin P Jordan, Richard A Hayward, Milisa Blagojevic-Bucknall, Peter Croft

**Affiliations:** Arthritis Research UK Primary Care Centre, Primary Care Sciences, Keele UniversityKeele, Staffs, ST5 5BG, United Kingdom

**Keywords:** cancer, musculoskeletal diseases, primary care

## Abstract

Musculoskeletal pain has been linked with subsequent cancer. The objective was to investigate associations between pain sites and specific cancers, and investigate the hypothesis that musculoskeletal pain is an early marker, rather than cause, of cancer. This was a cohort study in the General Practice Research Database. From a cohort of 46,656 people aged ≥50 years with a recorded musculoskeletal problem in 1996 but not during the previous 2 years, patients with a new consultation for back, neck, shoulder or hip pain in 1996 were selected and compared with 39,253 persons who had had no musculoskeletal consultation between 1994 and 1996. Outcome was incidence of prostate, breast, lung and colorectal cancer up to 10 years after baseline consultation. Strongest associations with prostate cancer were in the first year of follow-up for males consulting initially with back (adjusted hazard ratio 5.42; 95% CI 3.31, 8.88), hip (6.08; 2.87, 12.85) or neck problems (3.46; 1.58, 7.58). These associations remained for back and neck problems over 10 years. Significant associations existed with breast cancer up to 5 years after consultation in females with hip problems, and with breast and lung cancer in the first year after presentation with back problems. Previously observed links between pain and cancer reflect specific associations between pain sites and certain cancers. One explanation is liability for bony metastases from primary sites, and that pain represents a potential early marker of cancer. However, older patients with uncomplicated musculoskeletal pain seen in clinical practice have a low absolute excess cancer risk.

**What's new?**

Studies have suggested that musculoskeletal pain may be linked to cancer, though whether pain is an early symptom or a cause of cancer has remained unclear. In this study, new back, hip, and neck problems were found to be associated with the later diagnosis of prostate, breast, and lung cancer, mostly in the first year after baseline musculoskeletal consultation. However, risk of cancer is low and the association may be explained by liability of bony metastases from primary cancer sites, with pain being an early marker for disease.

Population-based studies from the UK have reported links between self-reported musculoskeletal pain and subsequent onset of cancer up to 8 years later, an association stronger for widespread than regional pain and for prostate and breast rather than other sites of cancer.^1^–^3^ A study based on a Finnish population though failed to confirm these findings.^4^

In our study of mortality in older adults following consultation for a new musculoskeletal problem in primary care, we confirmed an elevated risk of any cancer during 10 years of follow-up in these patients, but only in patients presenting with a back, hip, neck or shoulder problem.^5^ This pattern, in contrast to the population studies, suggests that such pains may be an early symptom of a developing cancer rather than some general component of chronic pain being a cause of cancer. A number of potential mechanisms could explain why musculoskeletal pain might occur before presentation of a primary cancer; these include micrometastases and paraneoplastic syndromes.^6^–^10^

We have now explored this hypothesis further in a study of incident cancers in this cohort of patients. We have investigated whether there are specific links between primary care consultations for new musculoskeletal problems and individual cancers, what happens to any such risks over time, and whether they could be explained by concomitant widespread pain.

## Material and Methods

The study was set in the General Practice Research Database (GPRD), a database of routinely collected primary care consultation data covering about 5% of the United Kingdom population. In the United Kingdom, primary care is commonly the point of entry into the health care system for people with a new symptom or illness and the major source of continuing care for chronic conditions. The accuracy of recorded morbidity codes in GPRD has been compared favorably to external sources such as secondary care information.^11^–^14^ The study was approved by the GPRD Independent Scientific Advisory Committee (ISAC) (project 06-069).

The study was based in general practices which contributed to the GPRD from before 1994 to after the end of 1996, and included patients who were continuously registered between these dates. Exposure was defined as at least one consultation during 1996 for a musculoskeletal problem with no such consultation in the previous 2 years, restricted to those aged 50 years or more at this baseline consultation in 1996. The definition of a musculoskeletal problem was based on the recorded Read or Oxmis code, used in United Kingdom primary care to record morbidities. These form a hierarchy of diagnostic codes which are grouped into chapters, for example, musculoskeletal or neoplasms. The unexposed comparison group drawn from the same database had no recorded musculoskeletal consultation at any time between 1994 and 1996 inclusive, and was matched by age, sex and practice to the musculoskeletal consulters. Some persons in the comparison group were matched to more than one musculoskeletal consulter, hence numbers are not identical. Baseline date was set at the date of first musculoskeletal consultation in 1996 or, for the comparison group, as January 1, 1997.

Those with a musculoskeletal consultation in 1996 were allocated to groups based on location of the problem (for example, back and knee) at their first musculoskeletal consultation in 1996. In this article, we have selected for detailed reporting only those groups identified in our previous mortality paper as having a link with any future cancer (back, neck, shoulder and hip).

Cancer was defined as a consultation for a malignant or pre-malignant neoplasm recorded under Chapter B of the Read Code hierarchy (“Neoplasms”) or the comparable Oxmis code. Benign neoplasms which are also coded in this Chapter were not included. For this new investigation we selected four of the commonest malignancies seen in primary care (prostate, breast, lung and colorectal cancer) and a research GP identified all codes related to these diagnoses. These four cancers include the two identified previously in the literature as being most strongly linked with prior complaints of pain (prostate and breast),^3^ and represent a mixture of cancers in which skeletal metastases are known to occur quite frequently (prostate and breast), less often (lung) or are very unusual (colorectal).^15^

### Statistical analysis

The follow up period for measuring cancer incidence was taken as a maximum of 10 years following baseline date. Those with a recorded cancer consultation in the 2 years pre-baseline were excluded from all analyses. Date of cancer was defined as its first recorded date. Analysis was performed separately for each of the four cancers: lung, prostate (males only), breast (females only) and colorectal.

Standardised cancer incidence ratios, indirectly standardised by age and gender, were calculated with 95% confidence intervals (based on the Poisson distribution) using the comparison group as the standard population. Persons leaving the study because of departure from their practice or departure of their practice from GPRD added their proportion of follow-up at time of leaving the study to the denominator. Cancer incidence rates over the first year are expressed per 10,000 person-years at risk. For the full (10 year) follow-up analysis, they are expressed per 10,000 person-decades at risk.

The exploration of relationship of location of musculoskeletal problem with the four specific cancers was developed by modelling the cancer rates for the four musculoskeletal groups and comparison group. The 10 years of follow-up was split into three time periods: (*i*) the first year after baseline, (*ii*) the second to fifth year and (*iii*) sixth to tenth year. Analyses were performed for the first year of follow-up on all persons, for the second to fifth year of follow-up in those with no cancer diagnosis by the end of the first year, and for the 6th to the 10th year in those with no cancer diagnosis by the end of the 5th year. Cox proportional hazards regression was used, with the comparison group as the reference group, with censoring at the earliest of last collected data point, point of death, departure from practice or the point at which the practice no longer contributed to GPRD. The proportionality assumption was assessed graphically and numerically, and deemed appropriate.

The models were adjusted for age, body mass index (BMI), smoking status, drinking status, deprivation and comorbidity. The rationale for this was the potential confounding effect of factors given they have been linked with both cancer and musculoskeletal conditions. Other potential confounders (notably physical activity) could not be included because they are not routinely recorded in primary care case-notes in the United Kingdom. The models for lung and colorectal cancer were also adjusted for gender.

For BMI, smoking and drinking status the measurement closest to baseline date was used where available. Each practice was allocated an Index of Multiple Deprivation score based on its geographical location to ascertain deprivation status of its local area.^16^ Comorbidity was defined as the number of diagnostic non-musculoskeletal Chapters (Read Code Chapters A–Z) for which patients had consulted in the 2 years prior to baseline: low (0–2 Chapters), medium (3–5 Chapters) and high comorbidity (6+ Chapters).

Finally, to assess whether any identified relationship between location group and incidence of cancer may be due to the concurrent presence of widespread pain, we carried out a final analysis excluding any persons in the exposed (back, shoulder, neck or hip) groups who subsequently had an episode of musculoskeletal pain during the first year of follow-up in a different location to the baseline recruitment episode. Previous work by our group on general practice data had established that recurrent consultation for pain in different musculoskeletal sites is a good proxy for the presence of chronic widespread pain,^17^ and so the group of exposed persons excluded from this final analysis were those with probable widespread pain. For example, if someone had consulted for a back problem on the baseline date and consulted for a knee problem in the first year of follow-up, they were removed from this final analysis.

Analysis was performed using Stata 12 for Windows and cia.^18^

## Results

As reported previously 48,206 persons with a new consulting episode of musculoskeletal problems were identified from 179 general practices, with 40,254 persons in the comparison group of persons who had not had such an episode.^5^ 1,550 persons with a new musculoskeletal problem and 1,001 from the comparison group were excluded on the basis of having a prior record of cancer. 37,561 (81%) of the 46,656 remaining persons with a new musculoskeletal episode consulted for a single, specified region. The back was the most predominant region (*n* = 8,929, 19%) and the hip was the least frequently recorded of the selected regions (*n* = 1,998, 4%). Median (IQR) length of follow up was 9.8 years (4.8, 10.0) for those with a new consulting episode of musculoskeletal problems and 9.4 years (4.5, 9.7) for the comparison group. The regional musculoskeletal groups were similar to each other, and to the comparison group, in terms of age, gender, and deprivation except that the hip group were older (mean age 70.4 years versus 65.7 for all new musculoskeletal consulters and 66.3 for comparison group) and more likely to be female [67% versus 56% (all new musculoskeletal consulters) and 55% (comparison group)].

The numbers of persons with a new diagnosis of the individual cancers are shown in Tables 1–4. Cancer rates expressed per 10,000 person years, and the standardised incidence ratios, overall and by the four selected regional groups, are also shown in Tables 4.

### Prostate cancer

The strongest relationship was found with prostate cancer in men. Those with any new musculoskeletal consultation in 1996 (standardised incidence ratio 2.98; 95% CI 2.43, 3.62) had significantly higher rates of prostate cancer in the first year of follow-up than the comparison group, and this was found for those presenting initially with three of the specific pain sites investigated: back (5.32; 95% CI 3.68, 7.43), hip (5.59; 95% CI 2.56, 11.32) or neck (3.53; 95% CI 1.52, 6.95) problems. These relationships were weaker but remained for the full 10 years of follow-up.

After adjusting for age, BMI, smoking and drinking status, deprivation and comorbidity, those presenting initially with a hip [hazard ratio (HR) 6.08; 95% CI 2.87, 12.85], back (HR 5.42; 95% CI 3.31, 8.88) or neck (HR 3.46; 95% CI 1.58, 7.58) problem had a significantly higher risk of prostate cancer in the first year of follow-up. Risk of new cancer diagnosis in these groups between years 2–5 was closer to that for the comparison group particularly for those with initial back problems but there was an increased risk of cancer between 6 and 10 years for those with initial back (HR 1.43; 95% CI 1.08, 1.90) or neck (HR 1.67; 95% CI 1.12, 2.46) problems (Table 5). There was a modest association with presenting initially with shoulder pain with prostate cancer after 5 years.

**Table 1:** Incidence of prostate cancer (men only) in 1st year and 10 years follow-up^1^

			Incidence in 1st year follow-up		Incidence in 10 years follow-up	
				
Group	*n*	Number with incident prostate cancer	Rate per 10,000 person-years	Standardised incidence ratio (95% CI)	Rate per 10,000 person-decades	Standardised incidence ratio (95% CI)
Comparison^2^	17654	414	18	1	327	1
Back	4062	142	87	**5.32 (3.68, 7.43)**	472	**1.60 (1.35, 1.89)**
Shoulder	1716	61	18	1.14 (0.24, 3.34)	463	**1.63 (1.25, 2.09)**
Neck	1485	59	55	**3.53 (1.52, 6.95)**	530	**1.80 (1.37, 2.33)**
Hip	665	31	144	**5.59 (2.56, 11.32)**	680	**1.66 (1.13, 2.35)**
All new musculoskeletal	20597	701	51	**2.98 (2.43, 3.62)**	461	**1.49 (1.38, 1.60)**

1 All incidence ratios were age standardised.

2 No musculoskeletal consultation in the 2 years prebaseline. Bold indicates *p*<0.05.

After removing the group of exposed patients with widespread pain, as per the definition in the methods, the relationship of hip problems with prostate cancer was no longer significant but remained for the back and neck groups (Supporting Information Table).

### Breast cancer

There was an increased incidence in the first year of breast cancer in women consulting with any new musculoskeletal problem in 1996 compared to the comparison group (standardised incidence ratio 1.49; 95% CI 1.18, 1.86). Women consulting initially with a hip problem had the highest rate of new record of breast cancer in the following year (2.59; 95% CI 1.04, 5.33), whilst those consulting initially with a back problem also had a higher incidence than the comparison group (2.12; 95% CI 1.31, 3.25). Incidence over the full 10 years was similar between the exposed and comparison groups (Table 2).

**Table 2:** Incidence of breast cancer (women only) in 1st year and 10 years follow-up^1^

			Incidence in 1st year follow-up		Incidence in 10 years follow-up	
				
Group	*n*	Number with incident breast cancer	Rate per 10,000 person-years	Standardised incidence ratio (95% CI)	Rate per 10,000 person-decades	Standardised incidence ratio (95% CI)
Comparison^2^	21599	500	21	1	321	1
Back	4867	134	46	**2.12 (1.31, 3.25)**	374	1.17 (0.98, 1.39)
Shoulder	1809	41	28	1.36 (0.44, 3.17)	298	0.97 (0.69, 1.31)
Neck	1753	42	23	1.13 (0.31, 2.90)	304	1.00 (0.72, 1.36)
Hip	1333	38	56	**2.59 (1.04, 5.33)**	448	1.29 (0.91, 1.76)
All new musculoskeletal	26059	635	31	**1.49 (1.18, 1.86)**	331	1.05 (0.97, 1.13)

1 All incidence ratios were age standardised.

2 No musculoskeletal consultation in the 2 years prebaseline. Bold indicates *p*<0.05.

In the adjusted model, the increased risk of breast cancer for women presenting initially with a back (HR 2.36; 95% CI 1.38, 4.01) or hip (HR 2.76; 95% CI 1.24, 6.16) problem was still evident in the first year (Table 5). The relationship disappeared after the first year of follow-up for those presenting initially with a back problem and after 5 years for those with a hip problem (Table 5). These relationships were still evident in the subgroups who only consulted for musculoskeletal problems in one region in the first year of follow-up (Supporting Information Table).

### Lung cancer

In the first year of follow up, there was an elevated incidence of lung cancer for all new musculoskeletal consulters compared to the comparison group (standardised incidence ratio 1.40; 95% CI 1.13, 1.70) and specifically for those consulting initially with a back problem (1.83; 95% CI 1.17, 2.72). However, incidence rates for the whole 10 years of follow-up were similar to the comparison group (Table 3).

**Table 3:** Incidence of lung cancer in 1st year and 10 years follow-up^1^

			Incidence in 1st year follow-up		Incidence in 10 years follow-up	
				
Group	*n*	Number with incident lung cancer	Rate per 10,000 person-years	Standardised incidence ratio (95% CI)	Rate per 10,000 person-decades	Standardised incidence ratio (95% CI)
Comparison^2^	39253	520	16	1	182	1
Back	8929	130	28	**1.83 (1.17, 2.72)**	195	1.15 (0.96, 1.36)
Shoulder	3525	44	23	1.51 (0.65, 2.97)	161	0.96 (0.70, 1.29)
Neck	3238	41	6	0.43 (0.05, 1.55)	163	1.00 (0.72, 1.36)
Hip	1998	24	16	0.86 (0.18, 2.51)	181	0.89 (0.57, 1.32)
All new musculoskeletal	46656	601	22	**1.40 (1.13, 1.70)**	173	0.99 (0.92, 1.08)

1 All incidence ratios were age standardised.

2 No musculoskeletal consultation in the 2 years prebaseline. Bold indicates *p*<0.05.

Following adjustment, the increased risk of lung cancer diagnosis in the first year of follow-up remained for those consulting initially with a back problem (HR 1.67; 95% CI 1.03, 2.70) but there was no increased risk after this first year (Table 6). When restricting the analysis to those consulting only for back problems during the first year of follow-up the HR fell slightly to 1.63 (95% CI 0.93, 2.85). (Supporting Information Table)

### Colorectal cancer

There was no relationship between new musculoskeletal consultations and colorectal cancer (Tables 4 and 6).

**Table 4:** Incidence of colorectal cancer in 1st year and 10 years follow-up^1^

			Incidence in 1st year follow-up		Incidence in 10 years follow-up	
				
Group	*n*	Number with incident colorectal cancer	Rate per 10,000 person-years	Standardised incidence ratio (95% CI)	Rate per 10,000 person-decades	Standardised incidence ratio (95% CI)
Comparison^2^	39253	438	10	1	154	1
Back	8929	96	14	1.42 (0.73, 2.48)	144	1.02 (0.82, 1.24)
Shoulder	3525	42	9	0.88 (0.18, 2.58)	154	1.10 (0.79, 1.49)
Neck	3238	40	6	0.67 (0.08, 2.43)	159	1.19 (0.85, 1.61)
Hip	1998	21	16	1.28 (0.26, 3.74)	159	0.87 (0.54, 1.33)
All new musculoskeletal	46656	514	11	1.06 (0.79, 1.40)	148	1.01 (0.92, 1.10)

1 All incidence ratios were age standardised.

2 No musculoskeletal consultation in the 2 years prebaseline.

**Table 5:** Adjusted hazard ratios (95% CI) for association of new musculoskeletal consultation with breast and prostate cancer

	Prostate cancer (men only) HR (95% CI)^1^			Breast cancer (women only) HR (95% CI)^1^		
		
Group	1 year FU	2–5 years FU	6–10 years FU	1 year FU	2–5 years FU	6–10 years FU
Comparison^2^	1	1	1	1	1	1
Back	**5.42 (3.31, 8.88)**	1.12 (0.80, 1.56)	**1.43 (1.08, 1.90)**	**2.36 (1.38, 4.01)**	1.12 (0.83, 1.51)	1.03 (0.77, 1.39)
Shoulder	1.12 (0.34, 3.68)	1.47 (0.96, 2.23)	**1.54 (1.06, 2.23)**	1.53 (0.60, 3.87)	1.16 (0.75, 1.80)	0.63 (0.37, 1.08)
Neck	**3.46 (1.58, 7.58)**	1.50 (0.96, 2.34)	**1.67 (1.12, 2.46)**	1.28 (0.46, 3.60)	0.81 (0.48, 1.37)	1.02 (0.66, 1.58)
Hip	**6.08 (2.87,12.85)**	1.45 (0.81, 2.61)	1.15 (0.61, 2.18)	**2.76 (1.24, 6.16)**	**1.88 (1.22, 2.89)**	0.62 (0.30, 1.25)

1 Adjusted for age, BMI, smoking status, drinking status, deprivation and comorbidity.

2 No musculoskeletal consultation in the 2 years prebaseline. FU = follow-up. Bold indicates *p*<0.05.

**Table 6:** Adjusted hazard ratios (95% CI) for association of new musculoskeletal consultation with lung and colorectal cancer

	Lung cancer HR (95% CI)^1^			Colorectal cancer HR (95% CI)^1^		
		
Group	1 year FU	2–5 years FU	6–10 years FU	1 year FU	2–5 years FU	6–10 years FU
Comparison^2^	1	1	1	1	1	1
Back	**1.67 (1.03, 2.70)**	1.12 (0.83, 1.51)	0.76 (0.56, 1.04)	1.34 (0.70, 2.59)	0.93 (0.66, 1.32)	1.00 (0.72, 1.38)
Shoulder	1.45 (0.69, 3.04)	0.93 (0.58, 1.49)	0.63 (0.39, 1.04)	0.85 (0.26, 2.75)	0.93 (0.56, 1.55)	1.14 (0.74, 1.76)
Neck	0.37 (0.09, 1.54)	0.69 (0.39, 1.21)	1.11 (0.73, 1.67)	0.65 (0.16, 2.69)	1.33 (0.84, 2.12)	1.04 (0.64, 1.70)
Hip	0.83 (0.26, 2.66)	0.60 (0.28, 1.27)	1.09 (0.63, 1.87)	1.25 (0.38, 4.07)	0.98 (0.52, 1.85)	0.80 (0.39, 1.62)

1 Adjusted for age, gender, BMI, smoking status, drinking status, deprivation and comorbidity.

2 No musculoskeletal consultation in the 2 years prebaseline. FU = follow-up. Bold indicates *p*<0.05.

## Discussion

This study has added to findings about musculoskeletal pain and future general risk of cancer and mortality by demonstrating relationships between new episodes of specific regional musculoskeletal problems in older persons attending primary care and subsequent onset of individual regional cancers. The associations observed in the first year of follow-up (back problems with lung, breast and prostate cancer; hip with breast and prostate cancer; and neck with prostate cancer) generally disappeared after the first year, although remained strong but reduced for prostate cancer up to 10 years after initial consultation.

An association between widespread pain and development of cancer up to 8 years later, particularly of breast and prostate, has previously been shown.^3^ We have previously found that new consulters presenting with single site problems in back, neck, hip or shoulder had a general increased risk of cancer diagnosis which was not evident for other pain sites.^5^ Here, we have added to this picture by showing that associations between specific musculoskeletal sites and different cancers did not include colorectal cancer; that the strength of these associations diminished with time; that the associations varied in strength for the different cancers—those with prostate cancer being the strongest and longest sustained; and that the presence of widespread pain did not explain the associations.

We constructed the cohorts to exclude all persons with a record of cancer in their medical case-notes in the 2 years prior to the onset of their musculoskeletal problem (and equivalent for the comparison group). Although it is possible that persons who had cancer earlier than this were not excluded, it is more likely that some record of it would have appeared in the case-notes during the 2 year period prior to recruitment and follow-up. Furthermore, the diagnosis and recording of cancer was made at a later date than the date of onset of the baseline musculoskeletal condition which defined recruitment into the exposure group; so all cancer diagnoses occurred subsequently to the initial musculoskeletal episode. One explanation of the patterns we observed is that musculoskeletal pain is an early marker of occult or developing cancer, prior to presentation and diagnosis of the primary cancer, rather than the alternative explanation that a general component of the mechanism or experience of chronic pain is a cause of cancer. The incidence of prostate cancer in the back group in the first year of follow-up was five times higher than the comparison group who had no musculoskeletal consultation at baseline. After the first year risk of cancer was similar (years 2–5) or only slightly higher (years 6–10) in those with initial back problems compared to the comparison group. It is possible that this may be partially explained by those in the comparison group becoming “exposed,” that is, consulting for a musculoskeletal problem during follow-up and prior to being diagnosed with cancer. Regardless, this reinforces the finding that the main impact is in the first year after a musculoskeletal consultation and the likely interpretation that the musculoskeletal problems are a marker rather than cause of cancer.

The pattern of risk observed (strongest for prostate and breast, no association with colorectal cancer) does mirror the pattern of risk of skeletal metastases. The locations of musculoskeletal pain carrying the strongest risk of future cancers (spine and hip) are among the most frequent sites of skeletal metastatic spread from prostate, breast and lung.^15^ Pain due to bony metastases can be the first presentation of prostate or breast cancer but usually only months before diagnosis and are likely to be due to the mechanical effect of the rapidly enlarging metastases in the bone.^6^

Micrometastases of prostate cancer in the spine may occur over 10 years prior to progression, whilst Ross reported the importance of both disseminated and circulating tumor cells to the prognosis in breast cancer.^19^,^20^ A review of micrometastases suggested they occur early in the life of a solid tumor and there may be a tendency for disseminated tumour cells to lodge in bone marrow.^7^,^21^ Two reviews concerning the relevance of disseminated tumour cells to progress of cancer concluded that tumour cells can lay dormant in bone marrow and for prostate cancer this may be for 10 years.^7^,^19^ It has not been reported that these micrometastases are related to pain.^8^,^9^,^22^ Metastases to bone in prostate cancer are common in several areas of the skeleton.^23^ Prostate cancer itself may lay dormant for many years, the trigger to why there is growth in some cancers but not others is uncertain.^24^–^26^ The importance of the micro-environment of the skeletal bone tissue in providing a stimulus for the seeding and growth of metastatic cancer has been highlighted in recent literature and may provide another explanation of musculoskeletal pain as a herald of future cancer.^27^

Certain rheumatic diseases are associated with the development of malignancy at sites in the body remote from the cancer (paraneoplastic syndromes).^8^–^10^ These paraneoplastic rheumatic disorders can precede the cancer by over 2 years and some bear similarities to premalignant conditions.^8^ Their aetiology is not clear but various mechanisms have been implicated including autoantibodies linked to cell death, inducing humoral and cell mediated immunity and release of cytokines.^8^,^9^ It is possible that some of the cancer diagnoses in this study might have fitted this category but there was no evidence from the available primary care records to confirm this.

There are other explanations of the observations, notably confounding by causal factors common to both musculoskeletal problems and cancer. We adjusted for potential confounders such as BMI and smoking which are routinely recorded in the medical records. It is possible that other confounders, such as physical activity, which were not included because they are not routinely recorded, could explain the findings, although the specific associations observed make this less likely.

The study has some potential weaknesses. It is possible that symptoms of cancer may be recorded elsewhere than under the Neoplasms Chapter. However, our study is based on definitive diagnoses of malignant and premalignant neoplasms. Furthermore, based on national cancer incidence figures for the United Kingdom, 17 new lung cancer cases per 10,000 population were expected in our comparison group in the first year. The actual figure of 16 per 10,000 suggests a comparable cancer rate in this group to the United Kingdom general population. We arbitrarily selected 2 years free of consultation for musculoskeletal problems as our definition of an incident case. Not all repeat consultations for chronic conditions in the GPRD need to be recorded.^28^ However, on-going consultations for the same problem should be recorded at least once during a 2 year period as the GPRD requires recording of morbidity if treatment changes. Morbidity codes are required to be entered at first diagnosis and hence incident cases should be comprehensively recorded.

This study has investigated problems in body locations rather than specific diagnoses. This is more relevant to the patient who will present initially with pain in a specific location, and may not obtain a clear diagnosis initially. Our assessment of widespread problems was based on primary care records and is not a stringent definition of widespread pain. There was some loss to follow-up but there is no reason to believe these patients were substantially different to those with longer follow-up. The groups had similar lengths of follow-up. The estimation of comorbidity ignores comorbidity occurring within the same Read Code Chapter, but reflects the range of comorbidities with which patients present.

With respect to the relevance of our findings for clinical practice, our previous study suggested that in a group of 1,000 new consulters to primary care aged 50 plus with back pain, there will be approximately 12 extra cases of cancer than would be expected in the next 12 months. Our new analysis suggests that these extra cases will be in the lung, prostate or breast. Prostate cancer is the most common but the absolute risk is small. Our study suggests prostate cancer will occur in the first year after presentation of back pain in one out of every 120 male new back pain consulters, and one out of every 75 male new hip pain consulters. Stratifying on age, prostate cancer will occur within the first year after presentation of back pain in one out of every 200 new male back pain consulters aged under 75 years and one in 40 aged 75 years or over. The equivalent figures for hip pain are one in every 156 new consultations (aged under 75) and one in 32 (aged 75 and over).

The absolute excess risk of cancer in patients presenting with common musculoskeletal problems is therefore low, and general practitioners and their patients can in general be reassured by these estimates from a large and representative national database of primary care consultations that new onset musculoskeletal pain is only rarely a harbinger of cancer. However, there is a need to ensure that primary care physicians, most of whom will know of such occasional rare possibilities and of patients' concerns about them, are also aware of the estimated size and nature of the increased risk for their discussions with older patients presenting with new back, hip or neck problems and to inform future consultations with those who return with other problems. Consultations for new back pain in the elderly are considered as red flag symptoms but this study shows that prostate cancer related to new hip pain is as common. Although the link between certain musculoskeletal conditions, such as hypertrophic osteoarthropathy, and cancer are recognised, most common musculoskeletal conditions seen in primary care are not closely related to cancer.^8^ As, we have discussed above, the reduction in risk over time suggests musculoskeletal problems are not a cause of cancer.

Regional musculoskeletal problems in the back, hip and neck are related to onset of new prostate, lung, and breast cancer in the elderly, particularly in those aged over 75 years, and in the first year after the initial musculoskeletal consultation, and one explanation is that these associations represent an early manifestation of the cancer. For prostate cancer, the link appears to extend for up to 10 years. However, the likelihood of cancer overall in older persons presenting in primary care with symptoms in these musculoskeletal sites is low, and the clear clinical message is that general practitioners and their patients do not, on this basis, need to search for cancer in the presence of uncomplicated back, hip or neck pain. Our study has provided specific estimates that can support discussion and reassurance in the consultation, and inform clinical practice if patients continue to consult with troublesome or additional symptoms. From the perspective of the science and biology of pain and cancer, further research is warranted into the mechanisms of these associations, including the possibility that they indicate early micrometastases to bone or an early marker of the potential for metastasis. Our study has found no evidence to support the hypothesis that musculoskeletal pain itself is a contributory cause of future cancer.

## Supplementary material

Supplementary Table: Adjusted hazard ratios (95% CI) for association of new musculoskeletal consultation with cancer for those consulting for problem in only 1 location in first year of follow-up
