# Supplementary material for: Incidence of prostate, breast, lung and colorectal cancer following new consultation for musculoskeletal pain: A cohort study among UK primary care patients
Source: Int J Cancer. 2013 Jan 25;133(3):713–20. doi: 10.1002/ijc.28055 (PMC3708122; doi:10.1002/ijc.28055)
Supplement: Supplementary file 1 [file ijc0133-0713-SD1.doc]

SUPPLEMENTARY TABLE: Adjusted hazard ratios (95% CI) for association of new musculoskeletal consultation with cancer for those consulting for problem in only 1 location in first year of follow-up

|  | PROSTATE CANCER (men only)  HR (95% CI)a | | | BREAST CANCER (women only)  HR (95% CI)a | | |
| --- | --- | --- | --- | --- | --- | --- |
| Group | 1yr FU | 2-5yrs FU | 6-10yrs FU | 1yr FU | 2-5yrs FU | 6-10yrs FU |
| Comparisonc | 1 | 1 | 1 | 1 | 1 | 1 |
| Back only | **4.06 (2.28, 7.23)** | 1.19 (0.83, 1.73) | **1.42 (1.04, 1.94)** | **2.26 (1.23, 4.17)** | 0.95 (0.66, 1.38) | 1.09 (0.78, 1.52) |
| Shoulder only | 0.53 (0.07, 3.90) | 1.31 (0.78, 2.19) | **1.65 (1.09, 2.48)** | 1.85 (0.66, 5.16) | 1.19 (0.71, 2.01) | 0.62 (0.32, 1.20) |
| Neck only | **2.91 (1.13, 7.54)** | 1.42 (0.84, 2.41) | 1.31 (0.80, 2.16) | 1.36 (0.42, 4.42) | 0.54 (0.25, 1.14) | 0.87 (0.49, 1.52) |
| Hip only | 3.01 (0.91, 9.91) | 1.20 (0.53, 2.72) | 0.71 (0.26, 1.92) | **3.26 (1.29, 8.26)** | 1.45 (0.79, 2.66) | 0.51 (0.19, 1.38) |

SUPPLEMENTARY TABLE (continued): Adjusted hazard ratios (95% CI) for association of new musculoskeletal consultation with cancer for those consulting for problem in only 1 location in first year of follow-up

|  | LUNG CANCER  HR (95% CI)b | | | COLORECTAL CANCER  HR (95% CI)b | | |
| --- | --- | --- | --- | --- | --- | --- |
| Group | 1yr FU | 2-5yrs FU | 6-10yrs FU | 1yr FU | 2-5yrs FU | 6-10yrs FU |
| Comparisonc | 1 | 1 | 1 | 1 | 1 | 1 |
| Back only | 1.63 (0.93, 2.85) | 0.90 (0.62, 1.30) | 0.79 (0.56, 1.12) | 1.76 (0.90, 3.46) | 0.85 (0.56, 1.29) | 1.16 (0.82, 1.64) |
| Shoulder only | 1.09 (0.39, 3.00) | 0.93 (0.53, 1.62) | 0.75 (0.44, 1.29) | 0.42 (0.06, 3.02) | 1.09 (0.62, 1.92) | 1.39 (0.88, 2.22) |
| Neck only | 0.28 (0.04, 2.01) | 0.75 (0.40, 1.43) | 1.31 (0.83, 2.05) | 0.91 (0.22, 3.79) | 1.48 (0.89, 2.47) | 1.21 (0.71, 2.06) |
| Hip only | 0.47 (0.06, 3.40) | 0.28 (0.07, 1.14) | 0.89 (0.42, 1.89) | 0.69 (0.09, 5.04) | 0.63 (0.23, 1.70) | 0.97 (0.43, 2.19) |

a adjusted for age, BMI, smoking status, drinking status, deprivation and comorbidity

b­ adjusted for age, gender, BMI, smoking status, drinking status, deprivation and comorbidity

c no musculoskeletal consultation in the 2 years pre-baseline.

FU = follow-up
